# Molecular mechanism of seed dormancy release induced by fluridone compared with cod stratification in *Notopterygium incisum*

**DOI:** 10.1186/s12870-018-1333-2

**Published:** 2018-06-11

**Authors:** Li Aihua, Jiang Shunyuan, Yang Guang, Li Ying, Guo Na, Chen Tong, Kang Liping, Huang Luqi

**Affiliations:** 1 State Key Laboratory Breeding Base of Dao-di Herbs, National Resource Center for Chinese Materia Medica, China Academy of Chinese Medical Sciences, Beijing, 100700 People’s Republic of China; 2 Flow Station of Post-doctoral Scientific Research, China Academy of Chinese Medical Sciences, Beijing, 100700 People’s Republic of China; 3 Sichuan Key Laboratory of Quality and Innovation Research of Chinese Materia Medica, Sichuan Academy of Chinese Medicine Sciences, Chengdu, 610041 People’s Republic of China; 4 Experimental Research Center, China Academy of Chinese Medical Sciences, Beijing, 100700 People’s Republic of China

**Keywords:** Abscisic acid (ABA), Cold stratification, Fluridone, Gibberellins (GA_s_), *Notopterygium incisum*, Seed dormancy release, Transcriptome, Strigolactones (SLs)

## Abstract

**Background:**

*Notopterygium incisum* is an important Chinese medicinal plant. Its mature seeds have underdeveloped embryos and are physiological dormant. We found the seeds with full developed embryos can germinate after treated by fluridone (FL), an inhibitor of abscisic acid (ABA). In order to understand the molecular mechanisms underlying seed dormancy release by FL, we compared the transcriptomic changes in dormancy release induced by two different methods, FL and cold stratification (CS) in *N. incisum*. We further analyzed the gene expression patterns involved in seed germination and dormancy using quantitative reverse-transcription PCR.

**Results:**

RNA-sequence analysis revealed more dramatic changes in the transcriptomes of FL than those in CS, particularly for genes involved in the biosynthesis and regulation of gibberellins (GAs) and ABA. The down-regulation of ABA biosynthesis genes and the dramatic up-regulation of *NiCYP707As*, an ABA catabolic gene, contributed to the reduced ABA levels in FL. The increased GA_3_ levels in CS-treated seeds were due to the up-regulation of *NiGA3OX*. Both *NiABI5* (a positive ABA regulator) and *NiGAI* (a negative regulator of GA) were down-regulated in FL and CS. The upregulation of strigolactones (SLs; the metabolites with the same precursor as ABA) biosynthesis and regulatory genes in both FL- and CS-treated seeds indicates that SLs contribute positively to seed dormancy release in *N. incisum*.

**Conclusions:**

Our results indicated that FL- and CS-seed dormancy release possibly depends on two totally different mechanisms: alleviation of the effects of ABA and potentiation of the effects of GA, respectively. However, NiABI5 and NiGAI probably function as common factors integrating the effects of ABA and GA on seed dormancy release.

**Electronic supplementary material:**

The online version of this article (10.1186/s12870-018-1333-2) contains supplementary material, which is available to authorized users.

## Background

The underground parts of *Notopterygium incisum* C. C. Ting ex H. T. Chang (Apiaceae) are used to produce the popular traditional Chinese medicine Qianghuo to treat headaches, the common cold, and rheumatism [1]. Seeds of *N. incisum* are morpho-physiological dormant (MPD), meaning that underdeveloped embryos must grow to a species-specific critical size and that physiological dormancy must be alleviated before seed germination [2]. However, little is known about the molecular mechanisms underlying seed dormancy release induced by FL and CS in *N. incisum*.

Abscisic acid (ABA) and gibberellins (GAs) are two main plant hormones involved in seed germination and dormancy via their ability to inhibit and promote germination [3]. Carotenoids are precursors of the ABA biosynthesis pathway [4]. Fluridone (FL) acts as an inhibitor of phytoene desaturase (PDS) activity, which converts phytoene to phytofluene, zeta-carotene, or neurosporene in the carotenoid-biosynthesis pathway [5–7]. It has been widely used as an inhibitor to prevent ABA biosynthesis [5], to relieve seed dormancy, and induce seed germination [8–11]. We found that FL treatment significantly improved seed germination in fully developed *N. incisum* seeds. Compared to treatment with CS for 3 months, a generally used method to release seed dormancy in *N. incisum* [12], the use of FL greatly reduced the seed germination period.

In order to investigate whether there is a common molecular mechanism between seed dormancy release induced by FL and CS in *N. incisum*, we used de novo transcriptomic analysis to find genes involved in the biosynthesis and regulation of ABA and GAs and their expression patterns.

## Methods

### Seed treatments and germination

*Notopterygium incisum* seeds were provided by Sichuan Notopterygium Organic Herbs Co., Ltd. (Aba prefecture, Sichuan province, China). Mature dry seeds were pretreated using the warm-cold moist treatment method [2]. The pretreated seeds were soaked in 100 mg/L FL solution for 24 h, with pure water used as the control (Con). CS treatment was carried out by storing the pretreated seeds in a sealed plastic box containing moist sand at 5 °C for 3 months. The seeds were removed from FL or sand (CS) and washed. The embryos were sampled from the washed seeds, frozen in liquid nitrogen immediately, and stored at − 80 °C for further analysis. To test the seed dormancy release, germination tests of CS, FL, and Con were carried out. Samples of 100 fresh seeds were sown on 1% water agar in 90 mm-diameter Petri dishes and incubated at 15 °C (the optimal germination temperature among the five temperatures tested; data not shown). The incubators were set to a photoperiod of 12 h light/ 12 h dark and 1000 lux white fluorescent light.

### RNA extraction, library preparation, and de novo sequencing

Total RNA was isolated from approximately 20 *N. incisum* embryos per sample using an RNeasy Plant kit (BioTeke Corporation, China). A total of 1.5 μg RNA per sample, with six samples from three treatments (Con, FL, and CS), was used for RNA sample preparation. The quality, purity and integrity of RNA were examined according to Ma et al. [13]. RNA library preparation was performed according to the protocol of the NEBNext® Ultra™ RNA Library Prep Kit for Illumina® (NEB, USA). Paired-end sequencing was carried out on an Illumina HiSeq 2500 platform.

### Gene functional annotation and differential expression analysis

BLAST analysis [14] was used for sequence annotation against seven databases, namely NCBI-Nr, NCBI-Nt, Pfam [15], KOG/COG, Swiss-Prot, KO, and GO, with expected E-values (E-value ≤1.0E-5 for Nr, Nt, and Swiss-Prot; E-value ≤0.01 for Pfam; E-value ≤1.0E-3 for KOG/COG; E-value ≤1.0E-10 for KEGG; E-value ≤1.0E-6 for GO). The expression levels of genes in each sample were assessed using RSEM [16]. The DESeq R package (1.10.1) was used for differential expression analysis [17]. To control the false discovery rate, the Benjamini and Hochberg’s approach was used to adjust the resulting *P* values. An adjusted *P*-value of < 0.05 was considered significant for differential gene expression.

### GO enrichment analysis and KEGG pathway enrichment analysis of the DEGs

Gene function classification of differentially expressed genes (DEGs) was performed using GO analysis with three categories including molecular function, cellular component, and biological process. The GOseq R package was used for GO enrichment analysis of the DEGs based on Wallenius’ noncentral hypergeometric distribution [18]. KOBAS software was used to test the statistical enrichment of DEGs in the KEGG pathway maps [19].

### Q-RT-PCR analysis

Total RNA was isolated from embryos using a Universal Plant RNeasy kit (BioTeke, China). Contaminating genomic DNA in the prepared RNA was removed using gDNA Eraser (Takara, Dalian, China) and the RNA was reverse transcribed using 1.0 μg of total RNA per reaction (20 μl) and the Prime-Script™ RT reagent Kit (Takara, China). Quantitative reverse-transcription PCR (q-RT-PCR) was carried out in an ABI 7500 system (Applied Biosystems, USA). The ABI Prism 7500 Sequence Detection System was used for data analysis according to Applied Biosystems User Bulletin. The expression levels of the genes in this study were normalized to that of endogenous control gene *NiGAPDH2*.

### ABA and GA_3_ measurements

ABA and GA_3_ levels were measured using ultra-performance liquid chromatography coupled with a quadrupole trap mass spectrometer equipped with electrospray ionization (UHPLC-ESI-MS/MS). ABA and GA_3_ were extracted using a revised method describe in [20]. Approximately 50 mg embryos were extracted from *N. incisum* seeds. UPLC separation was performed on a Waters Acquity UPLC-I-Class system (Waters Corporation, Milford, MA) with an Acquity BEH C18 column for chromatographic separation. The precursor-to-product ion transition was m/z: 263/153 for ABA and m/z: 345.1/143.1 for GA_3_. The LC-MS/MS system was controlled by Analyst 1.5.2, and the data analysis was performed using Multi Quantity software.

### Data analysis

All analyses were conducted with Microsoft Excel 2010 and Origin 7.0 software. The quantitative data are shown as means ± standard deviations. The data were subjected to one-way analysis of variance (ANOVA) with SPSS 16.0. Statistical significance was determined using Fisher’s least significant difference (LSD) test.

## Results

### Seed germination after FL and CS treatment and quantitative analysis of plant hormones in *N. incisum*

**Fig. 1:**
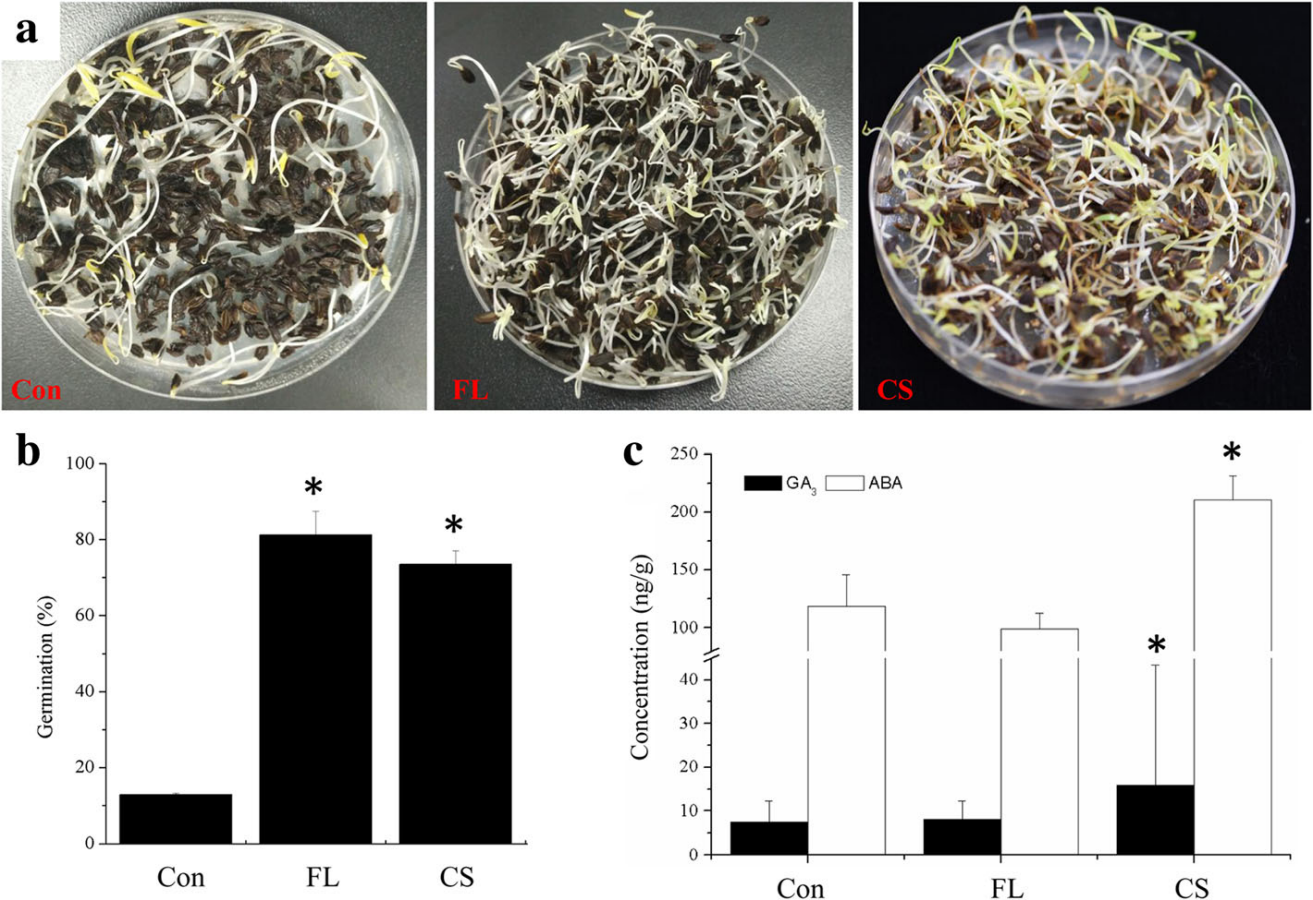
Germination and plant hormone levels in *N. incisum* seeds under different treatments. **a** Seed germination; **b** germination percentage; **c** plant hormone levels. Con, control; FL: imbibition with fluridone liquid for 24 h; CS: cold stratification for 3 months; the same in the following figures and tables. Data represent the mean ± SE (*n* = 3); “*” indicates a significant difference in FL or CS compared to Con at *P* < 0.05

Mature seeds of *N. incisum* have small, underdeveloped embryos, which must grow to ~ 2/3 of seed length during the warm, moist pretreatment period. Approximately 12.8% germination was obtained for untreated seeds (Con) with developed embryos (Fig. 1a, b). In contrast, about 73.5% germination percentage was achieved upon CS for 90 days, whereas 81.3% germination was obtained after the seeds were treated by FL (Fig. 1a, b). FL is an effective treatment for improving the germination of *N. incisum* seeds and can be used instead of cold stratification for 90 days.

We examined ABA and GA_3_ contents following three treatments with Con, FL, and CS via UHPLC-MS/MS. Both ABA and GA_3_ levels increased significantly in CS compared to Con (Fig. 1c). The accumulation of GA_3_ during CS is thought to be the key cause of seed dormancy release [21, 22]. FL reduces ABA levels to improve seed germination in several species [9, 10, 23]. ABA levels decreased from 118.5 ng / g to 98.7 ng / g after FL treatment in present work (Fig. 1c). GA_3_ level did not significantly change after FL treatment. These results suggest that sensitivity to ABA, not the ABA level, correlates directly with the dormancy status of *N. incisum* seeds, which is in agreement with other reports [23].

### Analysis of RNA-Seq datasets

Approximately 58, 60, 54, 55, 53, and 52 million raw reads were obtained from samples Con1, Con2, FL1, FL2, CS1, and CS2, respectively. The RNA-seq datasets were deposited in the Sequence Read Archive of NCBI (Access No. SRP107325). After filtering low-quality reads and adaptor sequences, 56.22, 59.02, 52.66, 53.94, 49.82, and 49.78 million clean reads and 8.43, 8.85, 7.9, 8.09, 7.47, and 7.47 G bases for samples Con1, Con2, FL1, FL2, CS1, and CS2 were obtained, respectively (Q30 of all samples ~ 90% and GC of all samples ~ 43%). In total, 18, 1376 unigenes with an average length of 650 bases and N50 of 939 bases were obtained using Trinity assembly software [24]. Sequencing and assembly information is provided in Table 1.

**Table 1:** Summary of the sequence assembly from *N. incisum* seeds

	Sample	Assembly size (n)	Total bases (bp)	GC (%)	Q30 (%)	Mean length (bp)	N50 (bp)
Clean reads	Con1	56,217,308	8.43G	42.84	89.21	–	–
	Con2	59,020,428	8.85G	42.89	89.34	–	–
	FL1	52,662,912	7.9G	42.83	88.87	–	–
	FL2	53,945,478	8.09G	42.74	90.77	–	–
	CS1	49,825,504	7.47G	42.7	91.74	–	–
	CS2	49,776,588	7.47G	43.25	92.34	–	–
Unigenes	All	181,376	117,946,891	–	–	650	939

BLASTX revealed that 68,236 of the unigenes (37.62%) significantly matched sequences in GO, KEGG, KOG/COG, NR, Nt, Pfam, and Swiss-Prot. The functional annotation for all genes in more detail is shown in Additional file 1: Table S1. Among the annotated unigenes, 51,594 (75.61% annotated in Nr) are predicted to be proteins. The gene sequences in *N. incisum* were most similar to those of *Vitis vinifera* (14.7%), followed by *Nicotiana tomentosiformis* (7.4%) and *Coffea canephora* (7.0%) (Additional file 2: Figure S1). The predicted unigenes were functionally categorized by GO annotation, with 39,353 unigenes assigned to GO classes (Additional file 3: Figure S2). The 17,942 unigenes were clustered into 26 functional groups by KOG analysis (Additional file 4: Figure S3). To help reconstruct the metabolic pathways in *N. incisum*, we performed KEGG pathway mapping for all of the unigenes. A total of 18,363 unigenes were successfully annotated and 281 KEGG pathways were mapped. Detailed pathway information is provided in Additional file 5: Table S2.

### Differential gene expression in FL and CS compared to con

**Fig. 2:**
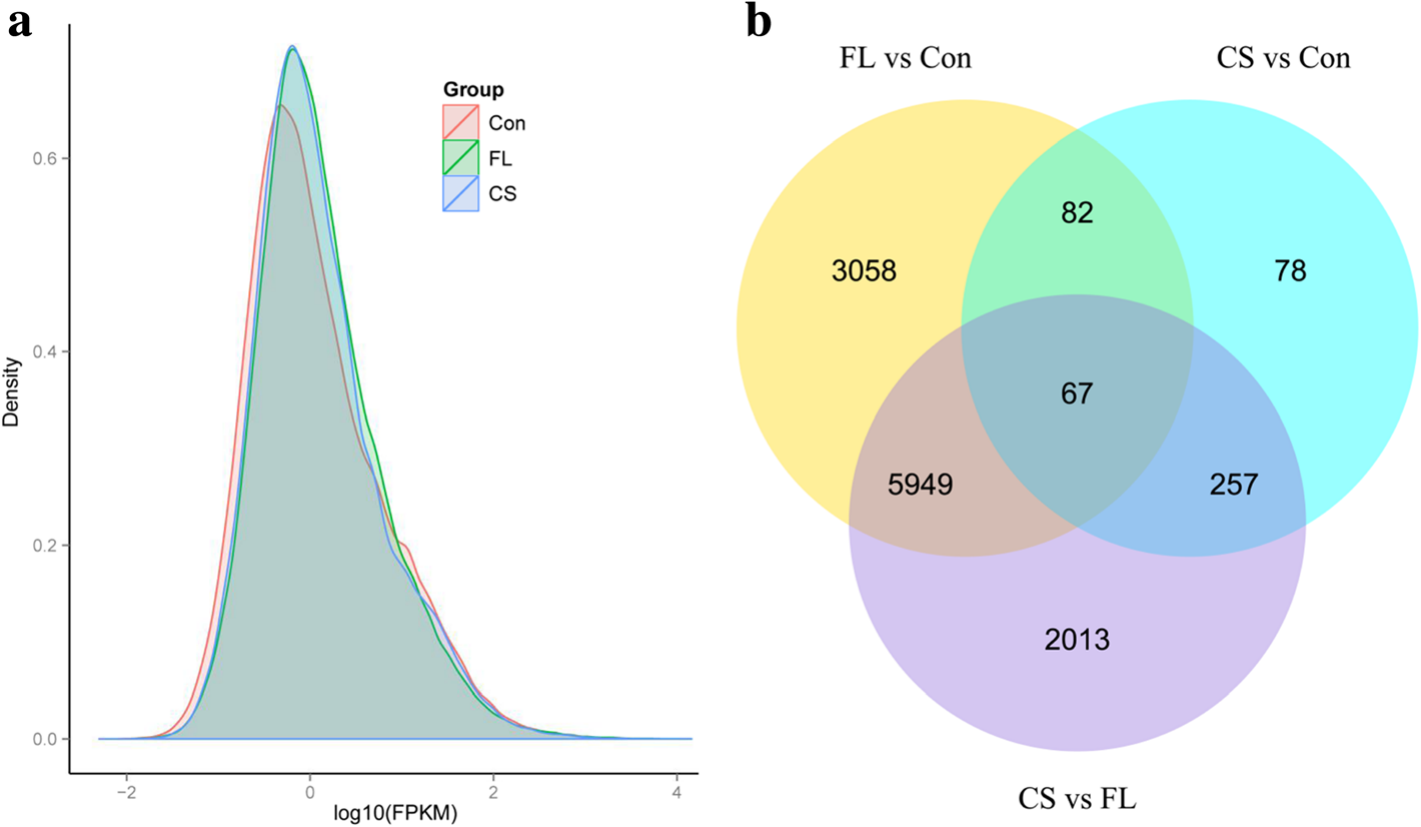
Differentially expressed *N. incisum* genes between Con, FL and CS. **a** FPKM density distribution; **b** Venn diagram

Based on the density plot of FPKM [25] values, the gene expression patterns of CS and FL were much more similar than those of Con and CS and Con and FL (Fig. 2a). These results are in agreement with the seed germination results, i.e., the seed germination percentage of CS and FL was higher than that of Con (Fig. 1). In total, 484 unigenes were differentially expressed between CS and Con, whereas 9156 DEGs were detected between FL and Con (Fig. 2b). There were 250 up-regulated and 234 down-regulated DEGs in CS, whereas there were 4142 up-regulated and 5014 down-regulated DEGs in FL. The top 20 enriched functional processes based on KEGG analysis between FL and CS compared to Con are shown in Additional file 6: Figure S4a, b. Among these processes, phenylalanine and phenylpropanoid metabolism were both highly enriched in CS and FL, as well as carbohydrate metabolism, amino acid and lipid metabolism, and genetic information processing. We also identified different enriched groups between FL and CS; for example, flavonoid metabolism (including flavonoid biosynthesis and flavone and flavonol biosynthesis) and photosynthesis processes are highly enriched in CS but not in FL (Additional file 6: Figure S4a). By contrast, the categories metabolism of xenobiotics by cytochrome P450, plant hormone signal transduction, and plant-pathogen interaction, and carotenoid biosynthesis were much more highly enriched in FL than in CS (Additional file 6: Figure S4b).

To elucidate the molecular changes that occur during seed dormancy alleviation in *N. incisum*, we clustered the DEGs expressed following CS and FL treatments by H-cluster and K-cluster analysis. H-cluster analysis showed that seeds of CS were much more similar to those of Con than to those of FL (Additional file 7: Figure S5a). Perhaps imbibition in FL solution is a much more crucial treatment than incubation in cold, moist conditions, as was performed during pretreatment. However, there were still some DEGs with similar expression patterns between FL and CS (Additional file 7: Figure S5a), which might be the key genes involved in seed germination and dormancy loss. K-clustering analysis identified six groups, including one group with 356 DEGs that were simultaneously down-regulated and one group of 114 ones that were simultaneously up-regulated in CS and FL (Additional file 7: Figure S5b). GO and KEGG enrichment analysis of the 468 co-expressed DEGs suggested that these DEGs function in numerous processes including mass metabolism (metabolic, proteolysis, carbohydrate metabolic, lipid metabolic, cellular metabolic, and cellular amino acid metabolic), energy metabolism (oxidation-reduction, NAD binding, and Rab GTPase binding), and genetic materials metabolism (nucleus, chromatin, chromatin binding, and nucleotidyl transferase activity) during seed dormancy release. The category “biological process of embryo development” was also enriched during this process, implying that genes involved in seed development are actively expressed (Additional file 8: Figure S6a).

We compared the biological processes occurring in FL vs. CS and found that most functional categories were more highly enriched in FL than in CS. However, flavonoid biosynthesis was much more highly enriched in CS than in FL (Additional file 8: Figure S6b). We identified ten key genes involved in flavone or flavonoid biosynthesis in *N. incisum* based on the RNA-Seq data and quantified their expression by reverse-transcription PCR (RT-PCR) using the primers listed in Table 2. Six of ten genes for key flavone biosynthesis enzymes, including phenylalanine ammonia-lyase 1 (*NiPAL1*), 4-coumarate: CoA ligase 2 (*Ni4CL2*), 4-coumarate: CoA ligase-like 5 (*Ni4CLL5*), chalcone synthase 2 (*NiCHS2*), p-coumarate 3-hydroxylase (*NiC3H)*, and flavone synthase I (*NiFNSI*), were significantly up-regulated in CS (Additional file 9: Figure S7). The dramatically increased expression of *NiPAL1* in CS suggests that secondary metabolites are biosynthesized in *N. incisum* seeds upon CS. The expression of *NiFNSI*, a flavone synthase gene particular to Apiaceae species [26], was significantly up-regulated in CS, but unchanged in FL (Additional file 9: Figure S7). The large number of up-regulated genes involved in flavone or flavonoid biosynthesis in CS suggests that one or both of these processes is induced during or after CS in *N. incisum*.

**Table 2:** Primers used in this study

Gene name	Unigene	Length of unigene (bp)	Primer
CCD8	c115090_g1	1836	5’ATTCATTCGGCTCATCCTAT5’TGTCCCTGGCTCCATTCTCA
D27	c124833_g3	3392	5’TTCATTCTTCTCCTCCTCTA5’GACGGTTTGCTACTTCTATT
C4H	c89387_g1	1840	5’TTGAGGCTAATGGAAATGAT5’CCAACCGTCCAATAGTGATA
4CL2	c114521_g1	2168	5’TGATCCTGATACGTCCATCT5’GCCAGCCTTCTTTGTCTATT
PAL3	c121330_g2	2578	5’GGATTATGGATTCAAGGGTG5’CTACTTGGCTTACGGTGTTT
CYP707A1	c119998_g1	1934	5’ATCGGAGAAACCTTCCAACT5’TAACACAAGGACATCCCAAT
ABI3	c125897_g1	4448	5’GTTGGGTGCTTCTGCTACTA5’TTTCTACACTAAACTTCCCT
ABI5	c126875_g2	1824	5’GCAGCCACAGTCACCACAGC5’CCCTTATCAGAAAGTCCTCG
ABF3	c125091_g2	3521	5’GTTACCTCGGACACTTAGCC5’TCACTCAAAGTTGCTTCCCT
GA2OX1	c101392_g1	1457	5’TGGTGAAACCCCAGAAAACT5’GGAACTGAAATCCAAGAGCC
GA20OX1	c64760_g1	1656	5’GGCGGTCTATTCGTCCTAAC5’TGGGCTCACCATCTTATCTT
GID1B	c104456_g1	2086	5’ACTTCTTCATCCATTCTTTG5’CTGGGTGGTCTCTATCTTCA
GASA14	c83558_g1	809	5’ACTGTGCCAAGTGAGGTGCG5’TGGGTGGTCATGTCGGTGTA
GASA6	c92944_g1	778	5’CACTTGGACAGGAACGGAAA5’TAGAAACCAGGGGGAACACA
LUT5	c123510_g1	3269	5’GGAGTCACTCTTTTCCCGTT5’CTATCTTCCGCTTCTCGCAG
PDS	c109548_g1	3109	5’CTCAATGGAGGGTGCTGT5’GGATTTATTTGGGTCGTA
VDE	c95526_g1	2509	5’GACAACTCGTGCATTTATTC5’TCCTTATCTACATTTATCCC
GAPDH2	c111413_g1	1948	5’CCGCCACCAGGTCTCATCTC5’GGGAACGGAAGCACCAAAGA
PAL1	c121330_g1	1560	5’CACATAAGTTGAAGCACCAC5’TTGACAGAATTGATCTCCCT
4CLL5	c112506_g1	1482	5’CAACTCACGGAAACCTAATC5’CAACTATCGGTGGCACTAAA
FAOMT	c116578_g2	1020	5’AAAGGAAGGGAGCATGAGTT5’CAAGGGCAGTAGTTAGGAGA
C3H	c107316_g2	2056	5’AGGAGCTTGACCGCGTAATC5’TGTGAGGGAGCATCATAGGG
CYP75B2	c109322_g1	2033	5’AGCCCTTACCCACTTCCACC5’ACCACCACATGAACCAACCC
FNSI	c102684_g1	1533	5’AGACCCGAGATATGCCGTAA5’TCGTCACCCTGAAGATGAGT
PYL5	c73037_g1	1771	5’AACAAATAGACGCCCCACTC5’TAACGCTAAAGCTCATCACA
CYP711A1	c125007_g2	4428	5’TTTACGCTATCCACAATCAT5’CTAACACTCCAAGAGCCAAC
DAD2	c111513_g1	1435	5’GTGTCCGTACCAGCATCAGT5’GCTCGTTAGCCAACAATCCC
MAX2	c48123_g1	2398	5’GGAATGTGGCTGACCTAACG5’AATCAATGTGAAGTCGCAAG
GA20OX5	c86483_g1	1756	5’AAAGCTGCACATGATGAAAT5’TCCGCATGAGCACCAGAGTA
GA3OX1	c79821_g1	2012	5’CGACTTGAATGACCCGATTA5’AGCTTCCTTGCAGCAACCTC
GA2OX2	c95152_g1	1338	5’TCAATAACGGTAGTGCTCCT5’TTGTCAAAGCCTGAAATGTG
GA2OX8	c113148_g1	1704	5’TTATCAACGGGCTTTCTACG5’AAACTGAAGGCCACCAACTA
GAI	c98921_g1	2363	5’GTTGAGCGACACGAGACATT5’CGAGTTGCCAAGCAGAGGTA
GID1C	c37750_g1	2004	5’CCAACGAGCCTACTTACCCG5’GTCCCGACTTTGAAGCCATG
RNA replication protein	c126998_g1	6214	5’GCCCACGGGTGAACTAATCT5’GTTGAGTGACATAGGCGAGA

We primarily focused on genes involved in the biosynthesis and signal transduction pathways of key plant hormones related to seed dormancy loss, such as GA and ABA, as well as strigolactones (SLs; metabolites derived from carotenoids) during FL and CS treatments in subsequent analyses.

### RNA-Seq data validation by RT-PCR

**Fig. 3:**
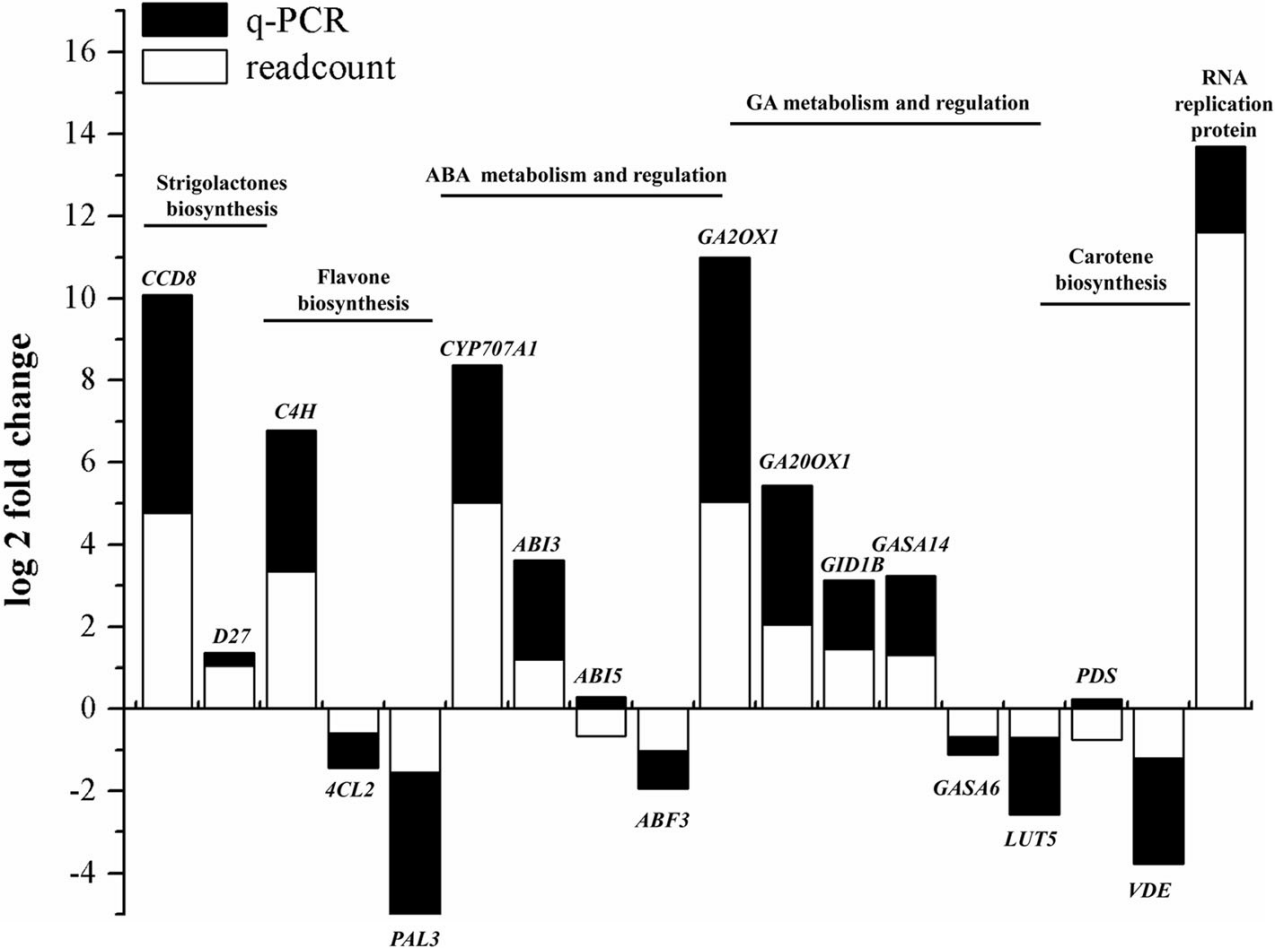
Comparison of relative gene expression levels detected by RT-PCR vs. RNA-Seq (read count). The data are log_2_-transformed values of the fold changes in FL compared to Con for *N. incisum* seeds

To validate the RNA-Seq results, we quantitatively assessed the expression patterns of the mRNAs using RT-PCR. We randomly selected 18 unigenes (some up-regulated, some down-regulated, and some with no change in expression; the primers listed in Table 2) involved in the biosynthesis and regulation of ABA, GA, SLs, and flavonoids. The gene expression patterns of the 16 unigenes revealed by RT-PCR agreed with those obtained by RNA-Seq (Fig. 3). However, the expression levels of *ABI5* and the metabolic enzyme gene *PDS* obtained by RT-PCR were different from those obtained by RNA-Seq, perhaps due to the lower level of accuracy of RNA-Seq, as observed in other studies [27]. In general, the RNA-Seq data confirm the gene expression patterns during the alleviation of seed dormancy in *N. incisum*.

### Gene expression of ABA and GA in FL

**Fig. 4:**
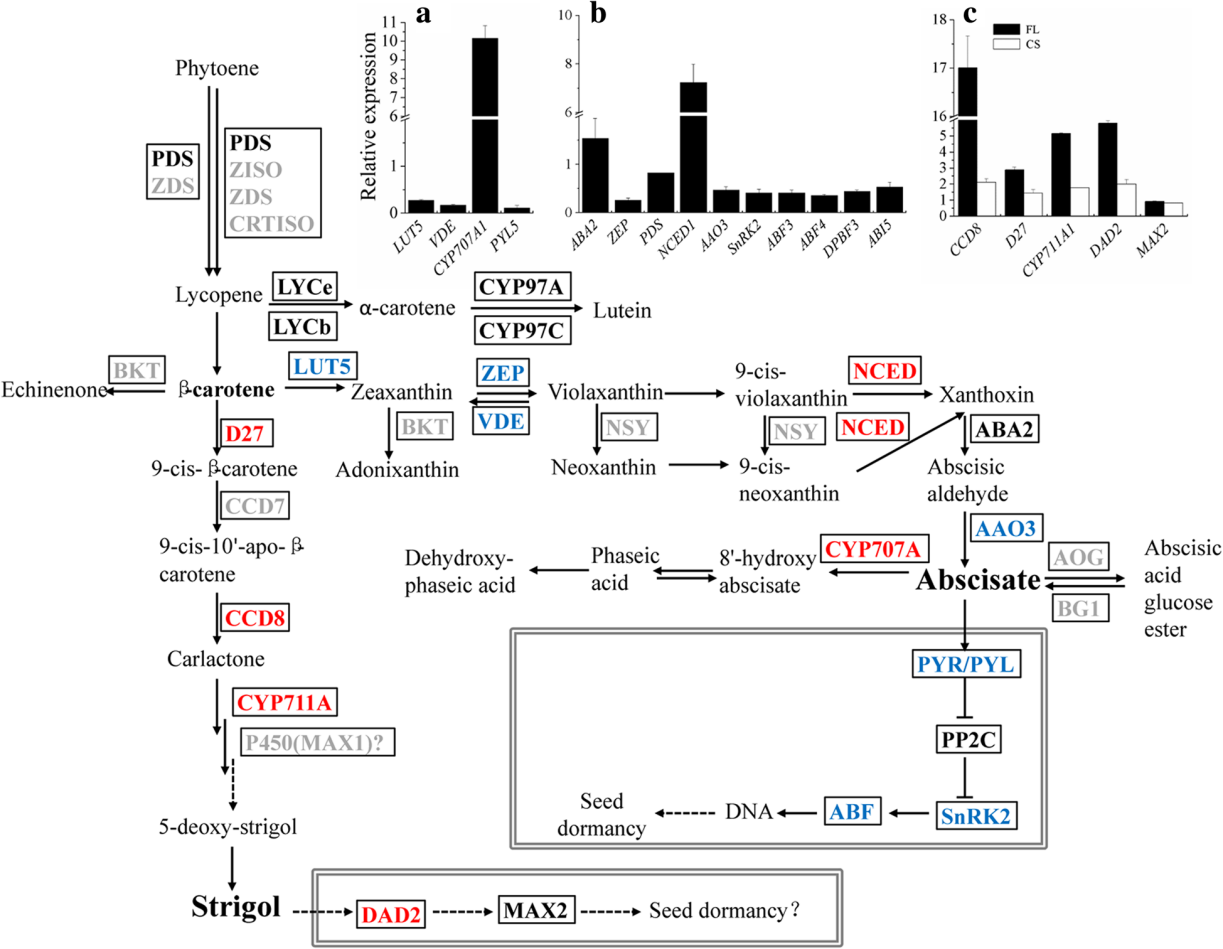
Metabolism and regulation of ABA and SL biosynthesis in *N. incisum* seeds. Up-regulated genes are shown in red, down-regulated genes are shown in blue, genes with no significant change in expression are shown in black, and genes not identified are shown in gray. The inset graphs show relative expression levels of the major ABA genes in FL compared to Con detected by RT-PCR (**a**) or RNA-Seq (**b**), and the major SLs genes in FL and CS compared to Con detected by RT-PCR (**c**). Data represent means ± SD (*n* = 3)

In total, 12 genes involved in ABA biosynthesis and signal transduction were identified in *N. incisum*. The *PDS* gene was slightly down-regulated after FL treatment (Fig. 4b, Additional file 10: Table S3). As were the genes of carotenoid beta-ring hydroxylase (*NiLUT5*), zeaxanthin epoxidase (*NiZEP*), and violaxanthin de-epoxidase (*NiVDE*) (down-regulated ~ 3.7-,3.8-, and 5.9-fold, respectively) (Fig. 4a, b, Additional file 10: Table S3). The subsequent downstream reaction involves the conversion of 9′-cis-violaxanthin or 9′-cis-neoxanthin to xanthoxin by 9-cis-epoxycarotenoid dioxygenase (NCED) [28]. Ten *NiNCED* genes were identified in the six *N. incisum* samples, eight of which were expressed at very low levels. In FL, the expression of *NiNCED1* and *NiNCED3* were up-regulated for 7.2- and 7.5-fold (Fig. 4, Additional file 10: Table S3). *NiABA2* was differentially expressed in FL (Fig. 4). The gene for aldehyde oxidase 3 (*NiAAO3*) catalyzed the last step of ABA biosynthesis [29] was down-regulated (~ 2.1-fold) in FL (Fig. 4, Additional file 10: Table S3). The expression of *ABA3*, encoding a MoCo sulfurase required by AAO3 for its activity [30], remained unchanged in FL (Additional file 10: Table S3). ABA 8′-hydroxylase (*CYP707A*) is involved in the degradation of ABA into phaseic acid [31]. Three genes, *NiCYP707A1*, *NiCYP707A4*, and *NiCYP707A7*, were significantly up-regulated in FL, especially *NiCYP707A1* (10.2-fold) (Fig. 4, Additional file 10: Table S3).

*NiPYL5*, the only differentially expressed ABA receptor gene, was down-regulated 8.9-fold in FL (Fig. 4, Additional file 10: Table S3). The gene for ABA-induced Snf1-related kinase 2 (*NiSnRK2*), a positive regulator of ABA signaling processes including stress tolerance and seed dormancy [32–35], was down-regulated ~ 2.5-fold in FL (Fig. 4, Additional file 10: Table S3). ABF/AREB subfamily genes *ABA-insensitive 5-like protein 6* (*NiABF3*), *NiABF4*, *ABA-insensitive 5-like protein 2* (*NiDPBF3*), and *ABA-insensitive 5* (*NiABI5*) were significantly differentially expressed (down-regulated ~ 2.4-, 2.8-, 2.2-, and 1.9-fold, respectively) in FL (Fig. 4b, Additional file 10: Table S3).

**Fig. 5:**
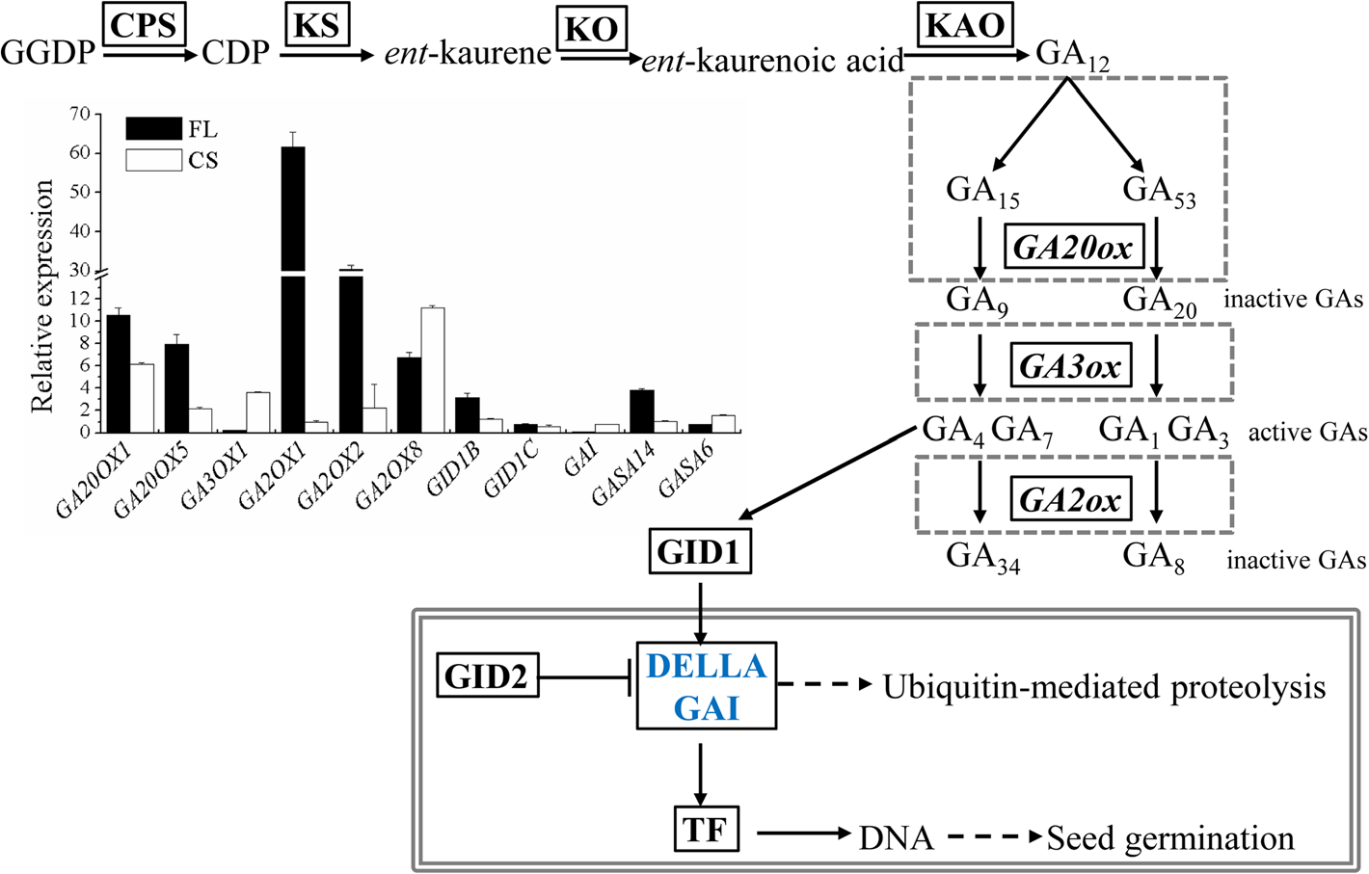
Metabolism and regulation of GA biosynthesis in *N. incisum* seeds. Genes down-regulated in both FL and CS are indicated in blue. The inset graph shows the relative expression levels of GA genes in FL and CS compared to Con detected by RT-PCR. Values are means ± SD (*n* = 3)

GA and ABA affect antagonistically seed germination and dormancy. GA improves seed germination. The formation of a C19-GA skeleton from C20-GA is catalyzed by GA 20-oxidase (GA20OX) [36]. The final step in the formation of physiologically active GAs is catalyzed by GA 3-oxidase (GA3OX) [37]. GA 2-oxidase (GA2OXs) is involved in the conversion from physiologically active GAs to inactive forms [38]. *NiGA20OX* was dramatically up-regulated (*NiGA20OX1* and *NiGA20OX5* were up-regulated 10.6- and 7.9-fold, respectively) in FL (Fig. 5, Additional file 10: Table S3). Gene *NiGA3OX* was down-regulated for 4.5-fold. Genes *NiGA2OX1*, *NiGA2OX2*, and *NiGA2OX8* were marked up-regulated for ~ 61.7-, 10.6-, and 7.9-fold, respectively (Fig. 5, Additional file 10: Table S3). Gibberellin receptor 1 (GID1) is a receptor of GAs whose accumulation is associated with seed dormancy loss in Arabidopsis [39]. *NiGID1B* was up-regulated 3.2-fold in FL, whereas the expression of *NiGID1C* in FL was similar to that of Con (Additional file 10: Table S3). The gene of DELLA protein gibberellic acid-insensitive (GAI), which represses physiological processes induced by GA [40], was down-regulated 10.5-fold in FL (Fig. 5, Additional file 10: Table S3). This is consistent with the observation that seed germination occurred after FL treatment. *NiGASA14*, the only differentially expressed GASA gene, was up-regulated 3.8-fold in FL (Fig. 5, Additional file 10: Table S3).

### Genes involved in seed dormancy release upon CS in *N. incisum*

Three genes *NiGA20OX1*, *NiGA20OX5*, and *NiGA3OX1*, encoding enzymes leading to the production of bioactive GA, were up-regulated 6.2-, 2.2-, and 3.6-fold, respectively in CS (Fig. 5, Additional file 10: Table S3). However, *NiGA2OX8*, which function in the inactivation of GA, were up-regulated as well (11.2-fold) (Fig. 5, Additional file 10: Table S3). *NiGAI* was down-regulated 1.4-fold in CS. It is positive related to seed germination. Among the GASA genes, although *NiGASA6* was significantly up-regulated (1.6-fold) in CS vs Con, *NiGASA9* and *NiGASA14* were not differentially expressed in CS (Fig. 5, Additional file 10: Table S3). Therefore, *NiGASA6* might be involved with seed dormancy loss induced by CS in *N. incisum.*

Although ABA levels increased significantly after CS (Fig. 1c), none of the ABA metabolism genes were differentially expressed, whereas *NiABI5* was significantly down-regulated (2.1- fold) (Additional file 10: Table S3).

### Gene expressions of SLs in FL and CS

SLs are produced from carotenoids in plants and play key roles in stimulating seed germination in parasitic plants [41, 42]. However, whether SLs are involved in seed germination in non-parasitic plants is currently unknown. Among the metabolites derived from phytoene during carotenoid metabolism, lycopene ε-cyclase (LYCe) and lycopene β-cyclase (LYCb) convert lycopene into α-carotene, and the latter is then converted into lutein by CYP97A/C [43]. We identified *NiLYCe*, *NiCYP97A*, and *NiCYP97C* in *N. incisum*, but they were not differentially expressed in FL or CS (Additional file 10: Table S3). The gene *NiLUT5* was down-regulated (3.7-fold) in FL but expressed at control levels in CS (Fig. 4a, Additional file 10: Table S3). D27, β-carotene isomerase dwarf 27, isomerizes all-trans-beta-carotene to 9-cis-beta-carotene, the first step in SL metabolism. Carotenoid cleavage dioxygenase 7 is responsible for the subsequent reaction, giving rise to the formation of 9-cis-beta-10′-carotenal, the latter being directly catalyzed into carlactone by CCD8 [44]. CYP711A functions in the conversion of carlactone to 5-deoxystrigol [45]. Among the four known genes involved in SL biosynthesis, three (*NiD27*, *NiCCD8*, and *NiCYP711A1*) were identified, and all were highly up-regulated (~ 2.9-, 17-, and 5.2-fold in FL vs. Con, and ~ 1.4-, 2.1-, 1.8-fold in CS) in *N. incisum* (Fig. 4c, Additional file 10: Table S3). By contrast, no gene encoding CCD7 was identified in any treated sample, but there might be other genes involved in the conversion from 9-cis-beta-carotene into 9-cis-beta-10′-carotenal in *N. incisum*. Moreover, no gene for BKT, which is involved in astaxanthin metabolism, was identified in *N. incisum* seeds. DAD2, a SL receptor, interacts with MAX2A in the presence of GR24 (a synthetic analogue of SLs) [46]. One *DAD2* and one *MAX2* gene were identified in *N. incisum* seeds. *NiDAD2* was highly up-regulated (5.8-fold in FL and 2.0-fold in CS), but *NiMAX2* was expressed at the level of the control in both FL and CS (Fig. 4c).

## Discussion

Upon FL, genes involved in ABA biosynthesis (*NiLUT5*, *NiZEP*, and *NiAAO3*) were down-regulated and genes involved in ABA catabolism (*NiCYP707As*) were dramatically up-regulated, indicating that these genes contributed to the reduction of ABA levels and degrading through hydrolyzation after FL treatment. The down-regulation of ABA receptor gene and its signaling regulator gene implied that the physiological events induced by ABA were retarded after FL treatment. Among them, genes *NiABF3*, *NiABF4*, *NiDPBF3*, and *NiABI5*, together with *NiPYL5* and *NiSnRK2*, were negatively correlated with seed dormancy release [47, 48].

The up-regulated *NiGID1B* suggests that *NiGID1B* might be important for seed dormancy loss, which also occurs when seed dormancy is broken by after-ripening and CS in Arabidopsis [39]. The dramatic down-regulation of DELLA protein gibberellic acid-insensitive (GAI) gene, which represses physiological processes induced by GA [40], is consistent with the observation that seed germination occurred after FL treatment. It indicates that GAI likely contributes to seed germination under FL treatment. GA-Stimulated Arabidopsis (GASA) proteins also play important roles in seed germination [49] and other developmental processes. Our results suggest GASA14 might play a positive role in seed dormancy release or seed germination in FL, and by positively affecting cell elongation during seed germination [50]. Although the expression patterns of GA metabolic genes implied that the level or activity of GA_3_ decreased after FL treatment, the expression of GA regulatory genes *NiGID1B*, *NiGAI*, and *NiGASA14* was positively involved in seed dormancy release treated by FL, and it might not strongly rely on GA_3_ levels or activity.

Dramatically up-regulation of three genes *NiGA20OX1*, *NiGA20OX5*, and *NiGA3OX1*, together with down-regulation of *NiGA2OX8* must attribute to the increase of GA level (Fig. 1c). Our results suggest that ABA regulators are involved in seed dormancy release induced by CS. The negative association of *NiABI5* with seed dormancy release in CS was the same as in FL. The up-regulation of *NiGASA6* and the down-regulation of *NiGAI* during CS suggest that they may be involved in CS-dormancy release.

Gene expressions of SLs biosynthesis and regulators suggest that SLs are actively synthesized after seed dormancy alleviation. Both the inhibition of ABA biosynthesis and the induction of SLs biosynthesis occurred after treated by FL in *N. incisum* seeds. The gene expression of SLs regulators indicates that SLs are active after FL and CS treatment in *N. incisum* seeds, further demonstrating that SLs might play a positive role in seed dormancy release in *N. incisum*.

Taken together, we propose FL- and CS-dormancy release involve completely different mechanisms involving the alleviation of ABA effects and potentiation of GA effects, respectively. Nevertheless, GA still positively regulated seed dormancy release in FL, and ABA regulators are involved in seed dormancy release induced by GA accumulation in CS as well. Moreover, down-regulation of *NiABI5* and *NiGAI* might occur or be necessary for seed dormancy release in *N. incisum*; these two genes possibly function as integrators of ABA and GA signaling associated with seed dormancy alleviation or germination.

## Conclusions

This study indicates that the processes involved in FL-dormancy release are much more complex than in CS-dormancy release. Different mechanisms occurred in FL- and CS-dormancy release: alleviation of ABA effects and potentiation of GA effects, respectively. Both ABA and GA regulators contributed to seed dormancy release irrespective of the dormancy treatment. It is possible that ABA and GA regulators act to integrate the effects of ABA and GA on seed dormancy release. The upregulation of SL biosynthesis and its regulatory genes in both FL- and CS-dormancy release imply that SLs are positive to seed dormancy release in *N. incisum*.

## Additional files

Additional file 1: **Table S1.** Summary of functional annotation of genes in *N. incisum* seeds. (XLSX 40668 kb)

Additional file 2: **Figure S1.** Gene expression patterns in *N. incisum* seeds aligned to those of other species in the Nr database. (TIF 6208 kb)

Additional file 3: **Figure S2.** GO classification of unigenes in *N. incisum*. The unigenes were assigned to the three GO categories: biological process, cellular component, and molecular function. (TIF 4228 kb)

Additional file 4: **Figure S3.** KOG analysis of unigenes in *N. incisum* seeds. (TIF 4024 kb)

Additional file 5: **Table S2.** KEGG classification of assembled unigenes in *N. incisum*. (XLSX 137 kb)

Additional file 6: **Figure S4.** KEGG enrichment analysis of DEGs in *N. incisum* seeds under FL (a) and CS (b) treatment compared to Con. (TIF 3608 kb).

Additional file 7: **Figure S5.** H-cluster (a) and K-cluster (b) analysis of differentially expressed genes in samples Con, FL, and CS of *N. incisum* seeds. (TIFF 5869 kb)

Additional file 8: **Figure S6.** GO (a) and KEGG (b) enrichment analysis of co-expressed DEGs in *N. incisum* seeds. BP, CC, and MF indicate tshe three GO categories: biological process, cellular component, and molecular function, respectively. (TIF 9584 kb)

Additional file 9: **Figure S7.** Relative expression of genes encoding enzymes involved in flavone or flavonoid metabolism in *N. incisum* seeds under FL and CS treatment compared to Con determined by RT-PCR. Data represent means ± SD (*n* = 3). (TIF 2011 kb)

Additional file 10: **Table S3.** The gene expression information of genes involved in ABA, GA, and SL biosynthesis and their expression under FL and CS treatments in *N. incisum* seeds. (XLSX 11 kb)

## Abbreviations

4CL2: 4-coumarate:CoA ligase 2
4CLL5: 4-coumarate:CoA ligase-like 5
AAO3: Aldehyde oxidase 3
ABA: Abscisic acid
ABF3: ABA-insensitive 5-like protein 6
ABI5: ABA-insensitive 5
C3H: p-coumarate 3-hydroxylase
C4H: Cinnamate 4-hydroxylase
CCD: Carotenoid cleavage dioxygenase
CHS2: Chalcone synthase 2
CS: Cold stratification
CYP707A: ABA 8′-hydroxylase
CYP75B2: Flavonoid 3′-monooxygenase
D27: β-carotene isomerase dwarf 27
DAD2: Decreased apical dominance 2
DEGs: Differentially expressed genes
DPBF3: ABA-insensitive 5-like protein 2
FAOMT: Flavonoid 3′,5′-methyltransferase
FL: Fluridone
FNSI: Flavone synthase I
FPKM: Expected number of Fragments Per Kilobase of transcript sequence per Millions base pairs sequenced
GA: Gibberrellin
GA20OX: GA 20-oxidase
GA2OX: GA 2-oxidase
GA3OX: GA 3-oxidase
GAI: Gibberellic acid-insensitive
GASA: GA-Stimulated Arabidopsis
GID1: Gibberellin receptor 1
LUT5: Carotenoid beta-ring hydroxylase
MAX: More axillary growth
MPD: Morpho-physiological dormant
NCED: 9-cis-epoxycarotenoid dioxygenase
PAL: Phenylalanine ammonia-lyase
PDS: Phytoene desaturase
PYL5: Abscisic acid receptor
SL: Strigolactone
SnRK2: Snf1-related kinase 2
UHPLC-MS/MS: Ultra-performance liquid chromatography coupled with a quadrupole trap mass spectrometer equipped with electrospray ionization
VDE: Violaxanthin de-epoxidase
ZEP: Zeaxanthin epoxidase
