# Supplementary figures and images for: Molecular mechanism of seed dormancy release induced by fluridone compared with cod stratification in Notopterygium incisum
Source: BMC Plant Biol. 2018 Jun 11;18:116. doi: 10.1186/s12870-018-1333-2 (PMC5996521; doi:10.1186/s12870-018-1333-2)

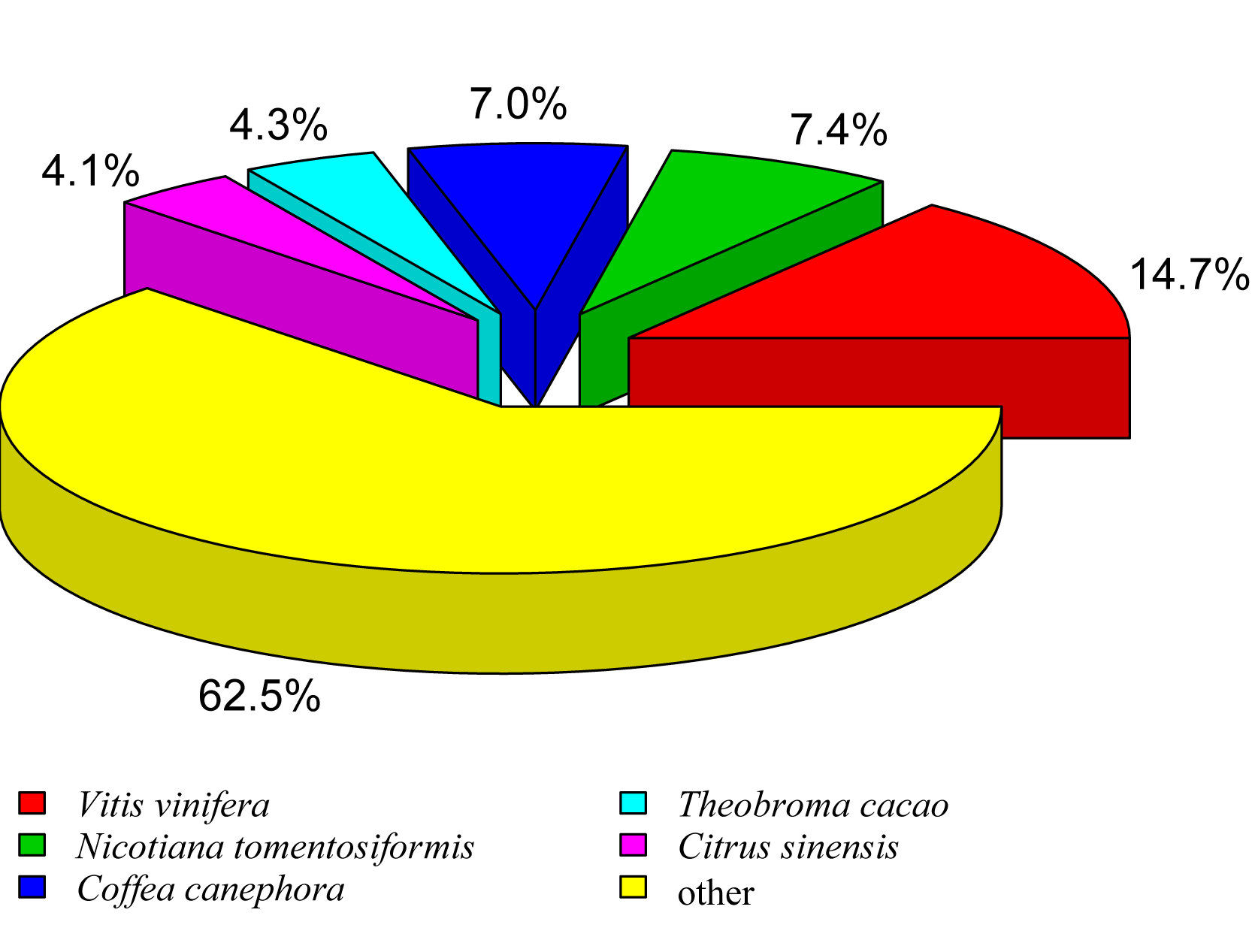

Supplement: Supplementary file 2 — Figure S1. Gene expression patterns in N. incisum seeds aligned to those of other species in the Nr database. (TIF 6208 kb) [file 12870_2018_1333_MOESM2_ESM.tif]

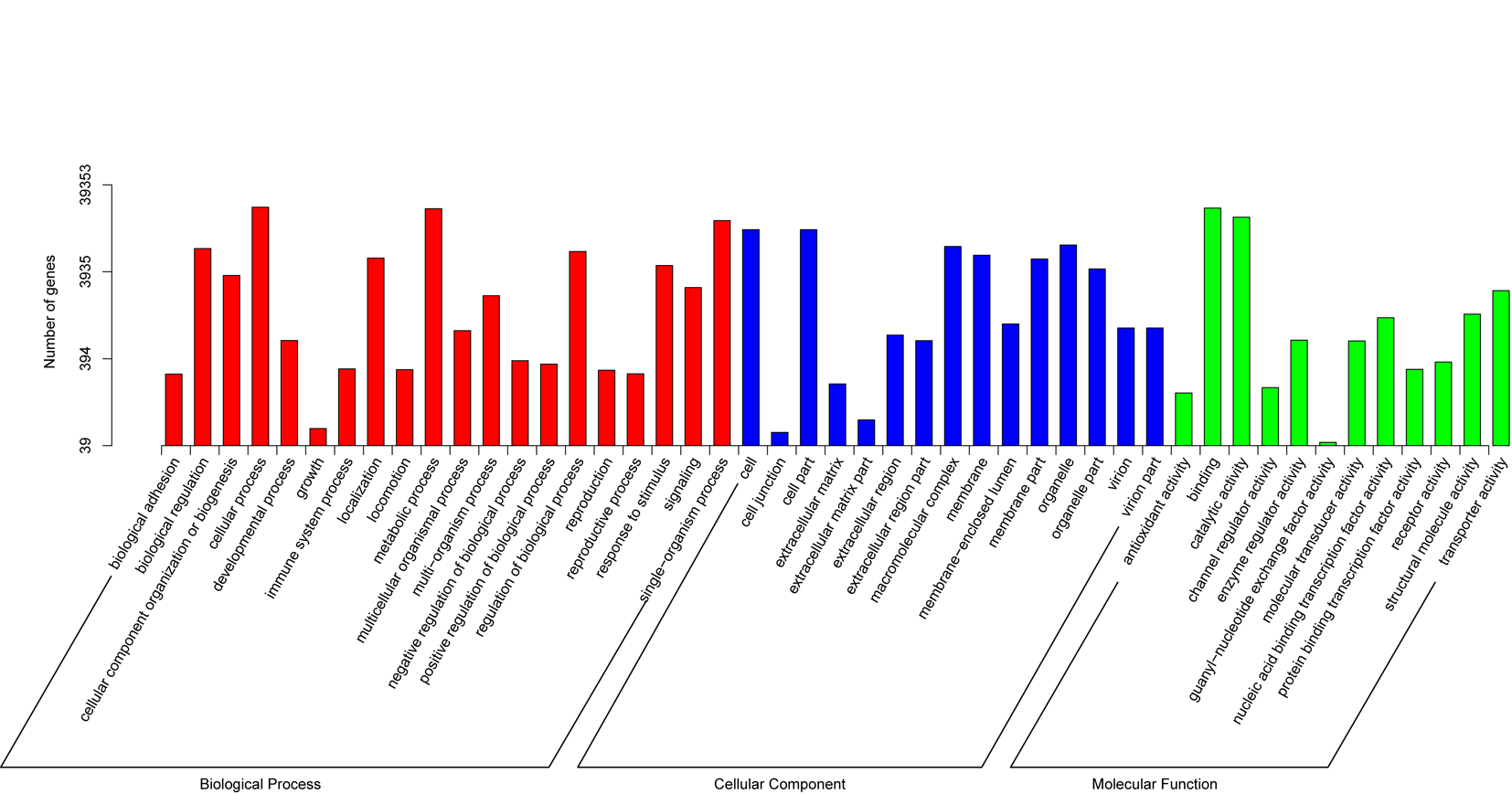

Supplement: Supplementary file 3 — Figure S2. GO classification of unigenes in N. incisum. The unigenes were assigned to the three GO categories: biological process, cellular component, and molecular function. (TIF 4228 kb) [file 12870_2018_1333_MOESM3_ESM.tif]

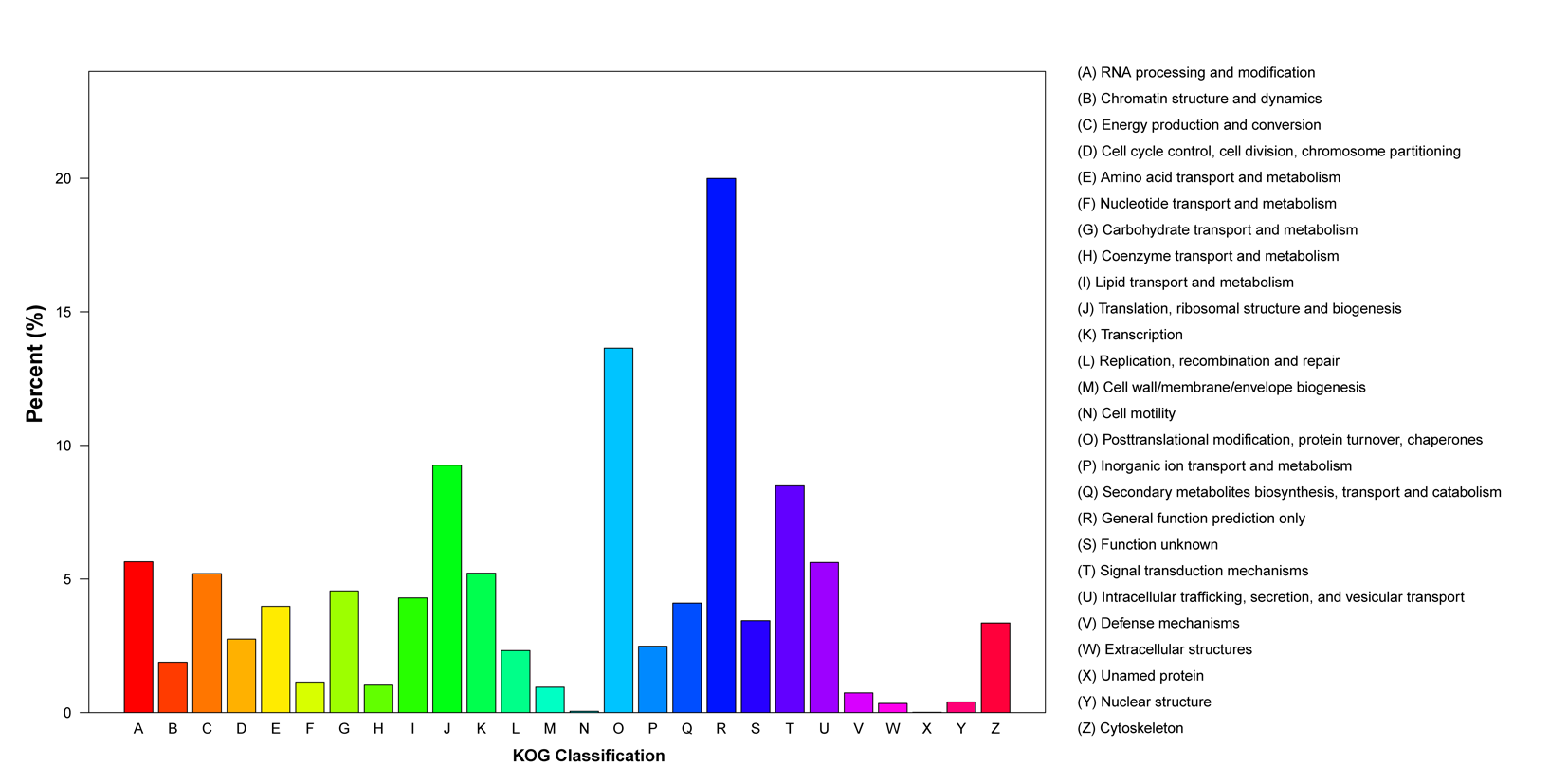

Supplement: Supplementary file 4 — Figure S3. KOG analysis of unigenes in N. incisum seeds. (TIF 4024 kb) [file 12870_2018_1333_MOESM4_ESM.tif]

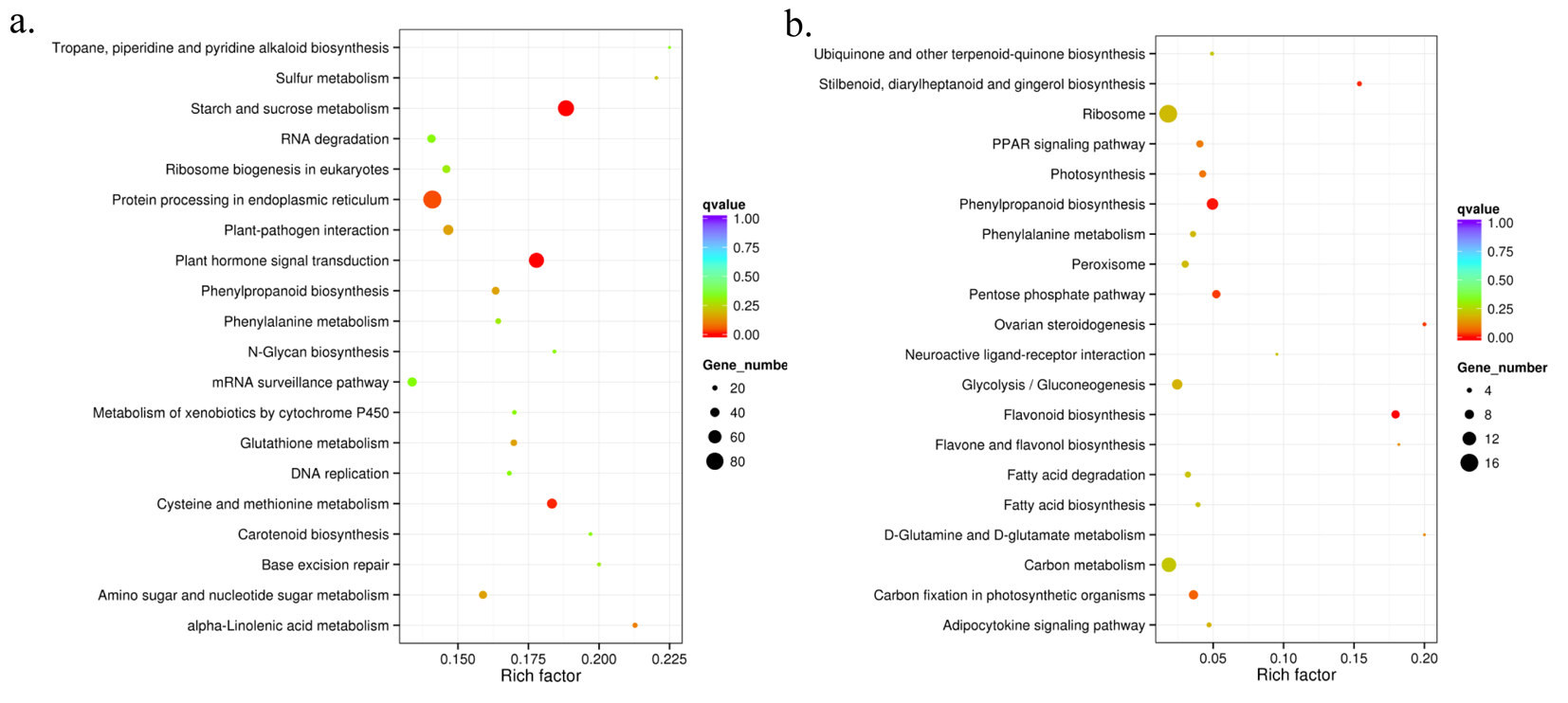

Supplement: Supplementary file 6 — Figure S4. KEGG enrichment analysis of DEGs in N. incisum seeds under FL (a) and CS (b) treatment compared to Con. (TIF 3608 kb). [file 12870_2018_1333_MOESM6_ESM.tif]

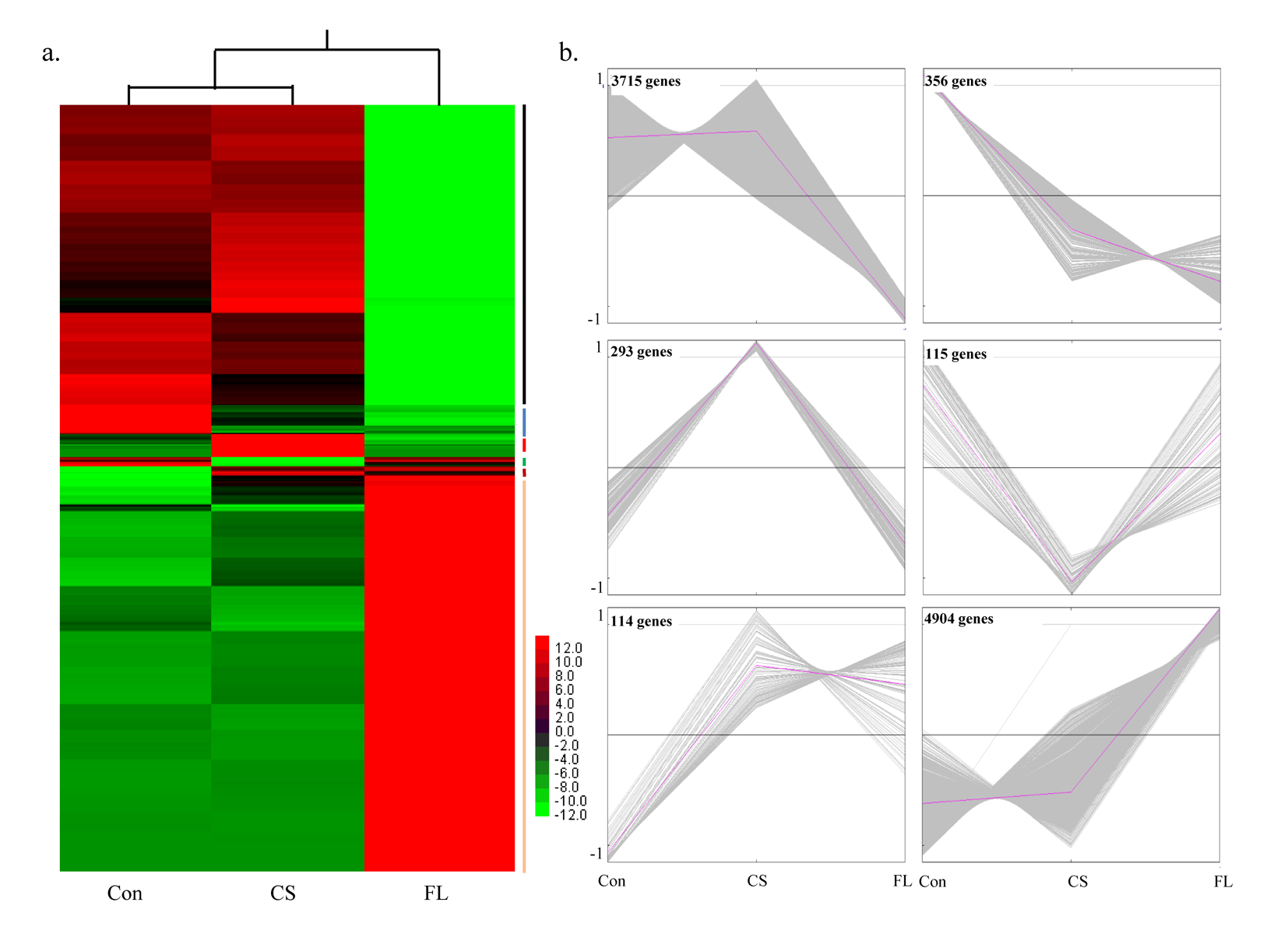

Supplement: Supplementary file 7 — Figure S5. H-cluster (a) and K-cluster (b) analysis of differentially expressed genes in samples Con, FL, and CS of N. incisum seeds. (TIFF 5869 kb) [file 12870_2018_1333_MOESM7_ESM.tiff]

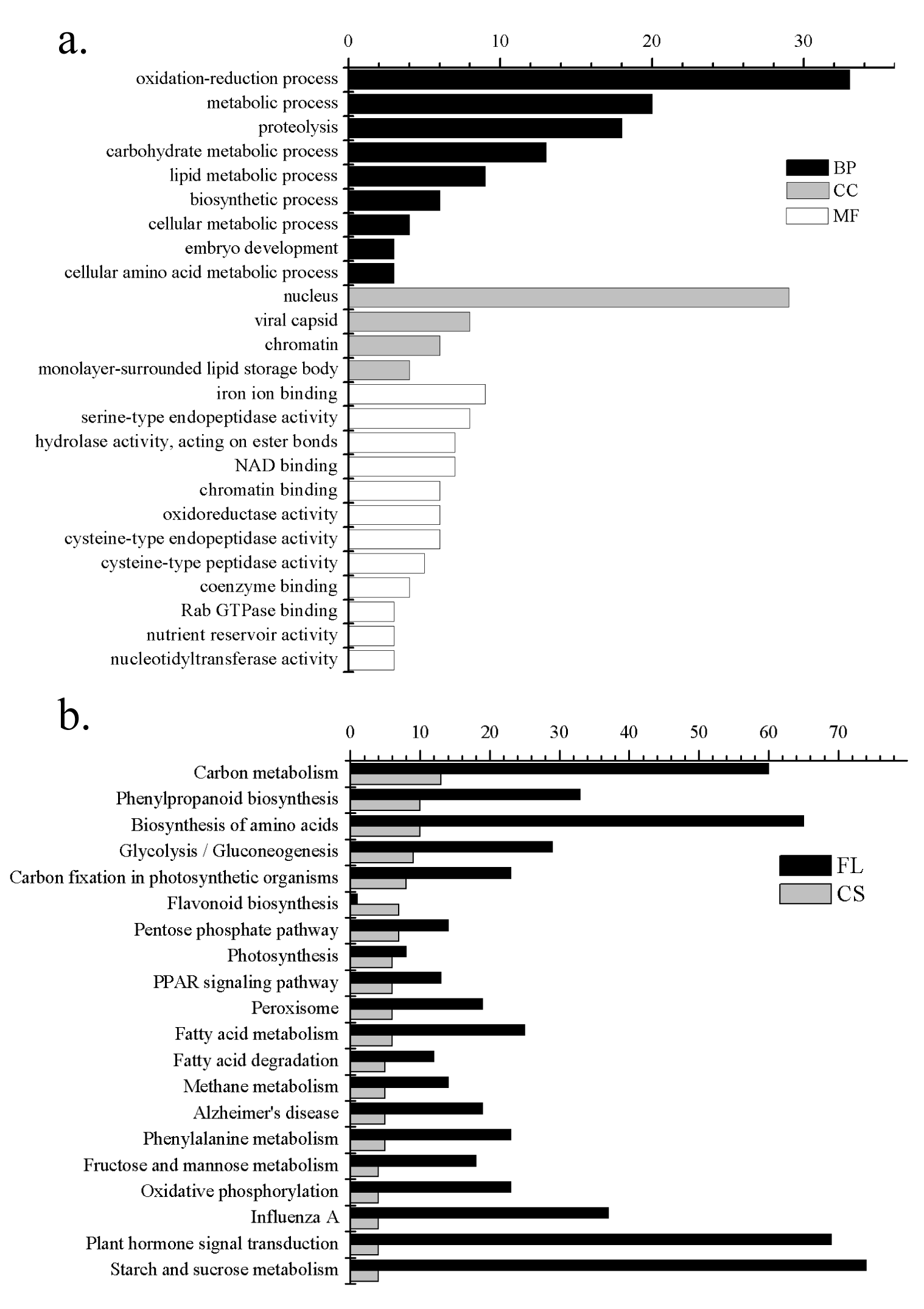

Supplement: Supplementary file 8 — Figure S6. GO (a) and KEGG (b) enrichment analysis of co-expressed DEGs in N. incisum seeds. BP, CC, and MF indicate tshe three GO categories: biological process, cellular component, and molecular function, respectively. (TIF 9584 kb) [file 12870_2018_1333_MOESM8_ESM.tif]
